# Hindrance of the Proteolytic Activity of Neutrophil-Derived Serine Proteases by Serine Protease Inhibitors as a Management of Cardiovascular Diseases and Chronic Inflammation

**DOI:** 10.3389/fchem.2021.784003

**Published:** 2021-11-15

**Authors:** Timo Burster, Zhadyra Mustafa, Dinara Myrzakhmetova, Anuar Zhanapiya, Michal Zimecki

**Affiliations:** ^1^ Department of Biology, School of Sciences and Humanities, Nazarbayev University, Nur-Sultan, Kazakhstan; ^2^ Hirszfeld Institute of Immunology and Experimental Therapy, Polish Academy of Sciences, Wroclaw, Poland

**Keywords:** serine protease inhibitors, serine proteases, neutrophil elastase, proteinase 3, cathepsin G, thrombosis, SARS-CoV-2, COVID-19

## Abstract

During inflammation neutrophils become activated and segregate neutrophil serine proteases (NSPs) to the surrounding environment in order to support a natural immune defense. However, an excess of proteolytic activity of NSPs can cause many complications, such as cardiovascular diseases and chronic inflammatory disorders, which will be elucidated on a biochemical and immunological level. The application of selective serine protease inhibitors is the logical consequence in the management of the indicated comorbidities and will be summarized in this briefing.

## Introduction to Serine Proteases and Functional Capacity

Proteases are categorized into six different groups, for instance, the serine protease class. These proteases are further subdivided to trypsin-like-, chymotrypsin-like-, and elastase-like serine proteases based on their preference of distinct amino acids at the P1 position, which is the position towards the N terminal end of the protein where hydrolysis of the sessile peptide bond occurs. Serine proteases cleave substrates irreversibly; therefore, their proteolytic activity is precisely regulated in three levels to circumvent serious adverse reactions (Korkmaz et al., 2008). First, proteases are expressed as zymogens, resident in special compartments (lysosomes or neutrophil granules), which then undergo post-translational modifications to become functional, and/or inhibited by endogenous natural inhibitors (serine protease inhibitors, serpins) (Burgener et al., 2019).

Serine proteases encompass the largest group of proteases, accounting for around 40% of all human proteases, which are crucial in health and disease (Di Cera, 2009). The catalytic triad of serine proteases depends on aspartate, serine, and histidine residues, whereby the oxygen atom of the hydroxyl group of the serine amino acid attacks the carbonyl group of the peptide bond located between P1 and P1’ of the target substrate and facilitates the proteolytical digestion of proteins or peptides (Kahler et al., 2020). However, when all checkpoints fail under certain physiological conditions, unlimited protease activity can lead to several diseases, indicating that the application of serine protease inhibitors controls unbalanced proteolytic activity to prevent comorbidities.

## Neutrophil Serine Proteases in the Cardiovascular System in Health and Disease

**FIGURE 1:**
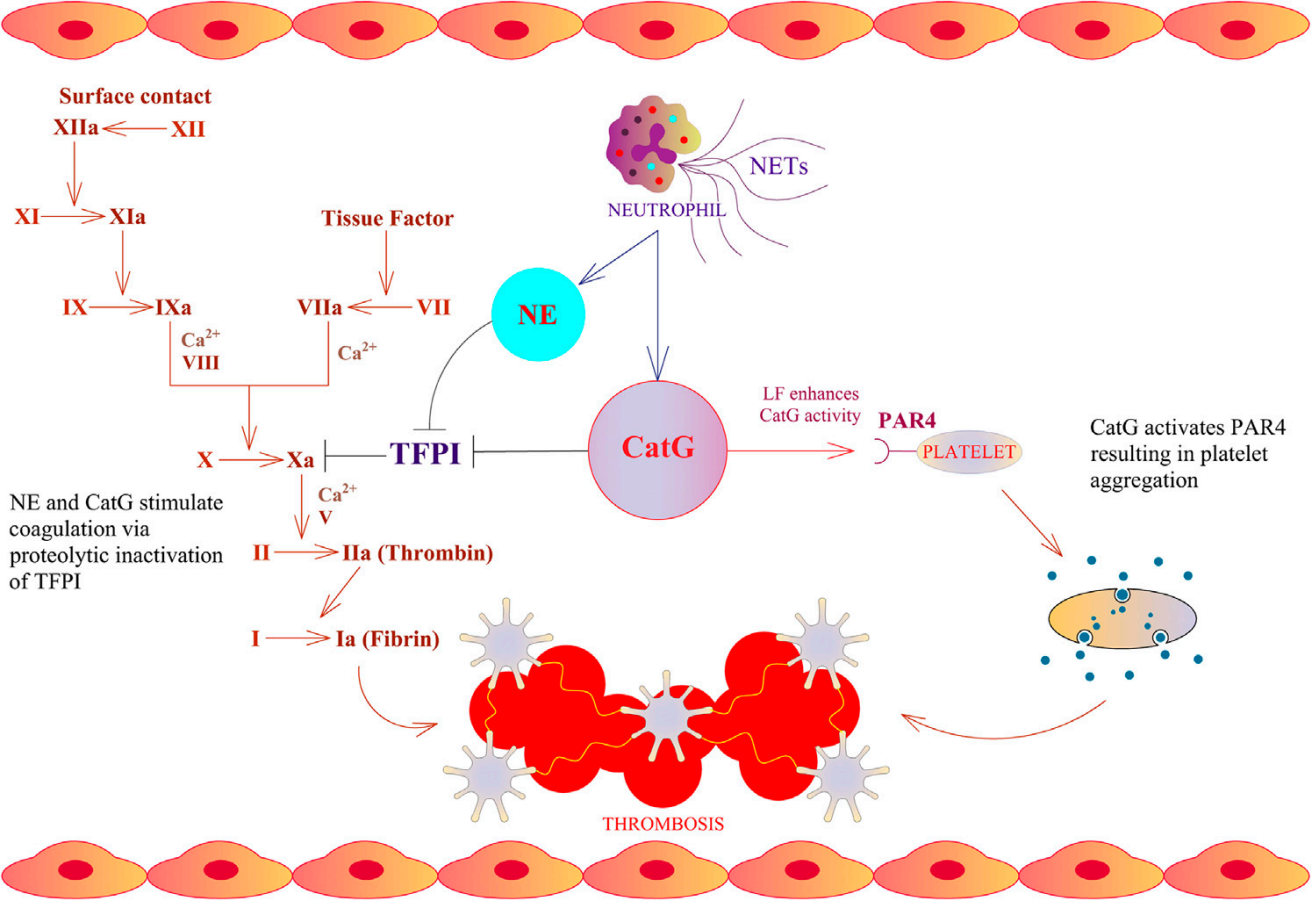
Thrombosis cascade and CatG. NE and CatG stimulate coagulation *via* proteolytic inactivation of tissue factor pathway inhibitor (TFPI), which is an endogenous inhibitor of factor X. CatG is able to stimulate platelet aggregation via cleavage of protease-activated receptor 4 (PAR4).

An immune response takes place after infection, initiating a first line of defense against invaders by the innate immune system. Particular neutrophils are in charge for the first line of defense. As a result, they strategically infiltrate the site of infection and release the content of granules. These granules contain serine proteases, encompassing neutrophil elastase (NE), cathepsin G (CatG), protease 3 (PR3), and neutrophil serine protease 4 (NSP4), which are collectively called neutrophil serine proteases (NSPs) (Korkmaz et al., 2010). Mast cells, resident in the tissue, secrete mast cell-derived tryptases, chymase, and CatG after activation stimuli (Korkmaz et al., 2019; Zamolodchikova et al., 2020). The accumulation of neutrophils and mast cells locally generates angiotensin II; thereby, secreted CatG and chymase hydrolyze angiotensin I selectively at phenylalanine 8 (F8) to produce angiotensin II which provokes vasoconstriction, regulates blood flow, and comprises an immune modulatory feature. These findings are in contrast to mouse CatG, where mouse CatG preferentially hydrolyzes at tyrosine 4 (Y4) and less prominently at F8. As a result, mouse CatG destroys angiotensin I rather than activates angiotensin II (Wintroub et al., 1981; Klickstein et al., 1982; Caughey et al., 2000; Raymond et al., 2010). Furthermore, angiotensin I can also be turned into angiotensin II by cell surface bound CatG on neutrophils, since cell surface bound CatG is remarkably resistant to inhibition by plasma born protease inhibitors, which indicates the important role of CatG in the local regulation of vasoconstriction by infiltrating neutrophils (Owen and Campbell, 1998). CatG hydrolyzes part of the tethered ligand from protease-activated receptor 4 (PAR4), which flips over to PAR4, activates platelets to form aggregates, initiates the coagulation cascade, and promotes thrombosis (Figure 1) (Sambrano et al., 2000; Heuberger and Schuepbach, 2019). Of note, LF further enhances CatG-mediated activation of platelets (Eipper et al., 2016). In the mouse model, tail bleeding time is extended by a CatG inhibitor as well as in CatG deficient mice, demonstrating reduced formation of neutrophil-platelet conjugates (Faraday et al., 2013). CatG and NE along with neutrophil-derived externalized nucleosomes (which form NETs) have been identified to stimulate coagulation via destruction and inactivation by proteolysis of the tissue factor pathway inhibitor (TFPI), which is an endogenous anticoagulant, resulting in arterial thrombosis (Massberg et al., 2010). On the other hand, CatG has an anti-coagulation capacity by cleaving factor V (Perrin et al., 2010).

CatG is not expressed in cardiac mast cells resident in healthy heart tissue; however, after heart failure, CatG can be detected in these cells (Jahanyar et al., 2007). Indeed, inhibition of both CatG and chymase in mice, which experienced coronary artery ligation and reperfusion, showed impaired levels of pro-inflammatory cytokines, lower levels of cardiac troponin, reduced cardiac caspase 3 activity, low pro-apoptotic Bax expression, as well as reduced number of macrophages, T cells, mast cells, and myocardial myeloperoxidase-positive neutrophils in the infarcted regions of the murine heart. These results indicate cardioprotective effects when both CatG and chymase are inhibited (Hooshdaran et al., 2017). Intracardial administration of CatG into rats marks high levels of pro-inflammatory cytokines and recruitment of neutrophils as well as macrophages to the myocardium (Miller et al., 2019). Additionally, neutrophil activation triggers the release of neutrophil extracellular traps (NETs), which encompass DNA and cytoplasmic granular proteins, like CatG, in order to catch and trap microorganisms in the extracellular space (Ma et al., 2019), resulting in enhanced inflammation, endothelial dysfunction, and induced thrombogenicity in a process depending on IL-1α precursor processing by CatG (Folco et al., 2018). In an early stage, CatG-deficient mice have an impaired wound healing capacity (Abbott et al., 1998) and neutrophil-derived CatG has been documented to promote myeloid cell adhesion to the arterial endothelium and contributes to atherosclerosis development, suggesting a partial blockade of CatG activity in patients with cardiovascular risk or concomitant inflammatory comorbidities (Ortega-Gomez et al., 2016).

NE plays a major role in neutralizing phagocytosed pathogens, exacerbates vascular permeability, attracts neutrophil migration to the site of the infection, and ensures the release of pro-inflammatory cytokines leading to inflammation (Korkmaz et al., 2008; Leinweber et al., 2021). Additionally, NE contributes to the removal of thrombosis by cleaving fibrin deposits that were already formed by platelets. On the other hand, NE promotes coagulation and thrombus formation by hydrolyzing the coagulation suppressor tissue factor (Massberg et al., 2010; Rabai et al., 2010). Therefore, inhibition of NE can be considered as a potential therapeutic approach for sepsis based on the fact that NE hydrolyzes proteoglycans which detach from the capillary vessel wall (Fukuta et al., 2020). Pulmonary arterial hypertension (PAH) is characterized by higher catalytic activity of NE hydrolyzing extracellular matrix components (elastin, laminin, collagen, and fibronectin) which in turn release epidermal growth factor (EGF) and fibroblast growth factor (FGF), leading to adverse vascular remodeling (Zhu et al., 1994; Taylor et al., 2018). NE-deficient mice possess an improved cardiac survival rate after myocardial infarction, indicating reduced inflammation and suppression of the Akt signaling pathway (Ogura et al., 2021). Of interest is the fact that administration of Sivelestat, a NE inhibitor, restored cardiac functioning post myocardial infarction in mice (Ogura et al., 2021). Moreover, elevated NE levels in plasma correlate with the severity of numerous cardiovascular diseases including coronary artery disease (CAD), ischemic heart disease, and angina pectoris (Mehta et al., 1989; Bell et al., 1990; Smith et al., 2000).

PR3 degrades proteins present in the extracellular environment, such as hemoglobin, fibronectin, laminin, elastin, collagen, and modulates the activity of both endothelial cells and thrombocytes (Csernok et al., 2008). Deficiency of PR3 correlates with an elevated prothrombotic risk in patients with paroxysmal nocturnal hemoglobinuria (Jankowska et al., 2011). Similar to NE, increased levels of PR3 are linked with a decline in survival rates post-myocardial infarction in patients (Bell et al., 1990).

In the case of NSP4, an arginine-specific neutrophil serine protease, this protease can regulate mast cell-dependent vascular leakage by mediating the proper maturation of secretory granules and subsequent storage of vasoactive amines (histamine and serotonin). In NSP4-deficient mice the morphology of secretory granules of mast cells was irregular with reduced levels of serotonin and histamine leading to a decrease in the amount of vascular leakage (AhYoung et al., 2020).

## The Role of Neutrophil Serine Proteases in the Respiratory System

Acute lung injury, chronic obstructive pulmonary disease (COPD), and acute respiratory distress syndrome (ARDS) are considered to be the result of neutrophilic inflammation of the lung (Polverino et al., 2017; Matthay et al., 2019). The imbalance between protease activity and endogenous protease inhibitors is a result of protease-mediated pathogenesis. Indeed, dysregulated protease activity provokes the upregulation of pro-inflammatory mediators resulting in excessive inflammation, attraction of immune cells, degradation of antimicrobial peptides and proteins, and destruction of lung tissue, resulting in COPD (Lucas et al., 2013). A logical consequence is to treat COPD with protease inhibitors with the characteristics of a long-term application to improve the impact of protease inhibitor treatments. Additionally, it is important to identify a key protease(s) crucial for direct tissue destruction and specific interference with the proteolytic activity of such a protease(s) instead of using an unspecific inhibitor or a panel of different inhibitors (Dey et al., 2018). A high number of proteases are present in the COPD lung; of these, all four mechanistic classes of proteases, serine-, cysteine-, aspartic-, and matrix metalloproteases, are part of the pathogenesis of COPD. Serine proteases, such as NE, PR3, CatG, dipeptidyl peptidase 4, and chymase, are some of those associated with the severity of COPD (Korkmaz et al., 2010). Here we will mainly focus on NSPs which are involved in COPD.

Although CatG protects against Streptococcus pneumonia-induced lung damage, high expression of CatG causes alveolar wall destruction and genetic ablation of CatG is protective when facing lung tissue damage caused by cigarette smoke (extensively reviewed in Dey et al. (2018)). Activated neutrophils and mast cells tend to accumulate in inflamed airways and lungs and these cells release proteases, including CatG, leading to extracellular matrix degradation as well as lung tissue damage and remodeling. Administration of nebulized RWJ-355871, which is a potent non-peptide, small-molecule inhibitor of CatG and chymase, to chronically tobacco smoke-exposed mice reduced the neutrophil burden by 66% in the bronchoalveolar lavage fluid (BALF) in contrast to control mice (Maryanoff et al., 2010). Furthermore, genetic knockdown of CatG, NE, and PR3 shields against lung tissue destruction and emphysema development after long-term exposure to cigarette smoke, suggesting that CatG, NE, and PR3 are involved in the degradation of lung connective tissue (Guyot et al., 2014).

**FIGURE 2:**
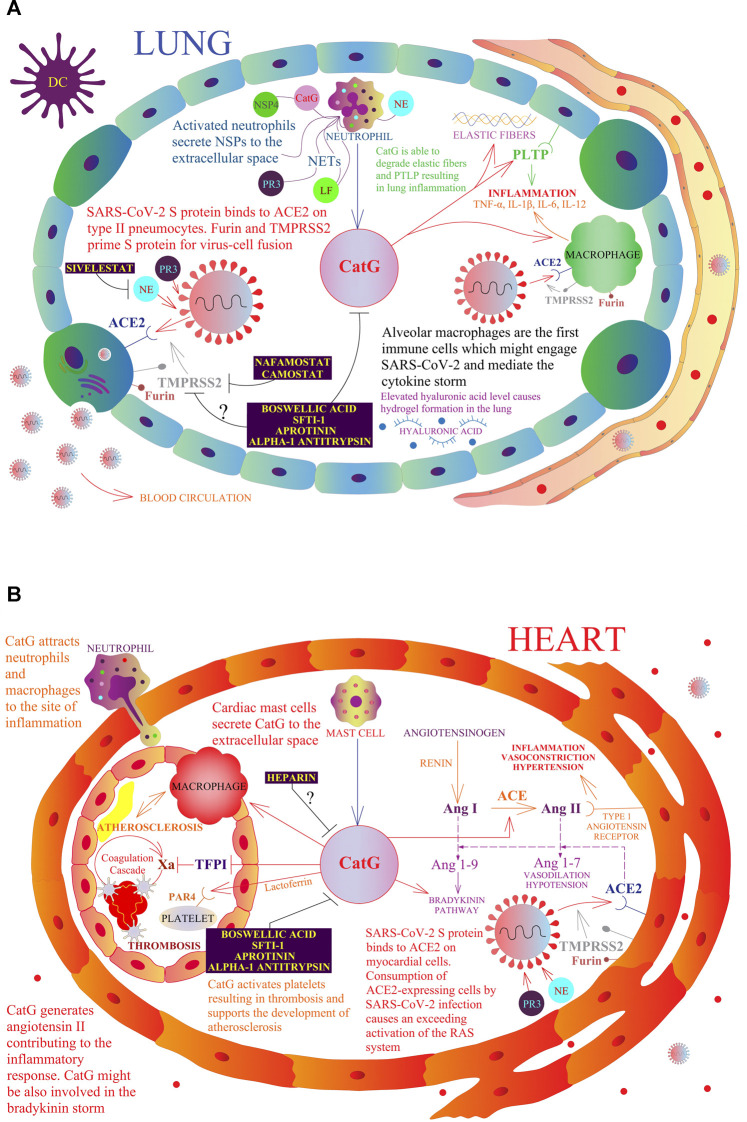
A model of a possible role of NSPs in the respiratory and cardiovascular systems in the context of COVID-19. The multivariate role of CatG in the human organism, including promotion of inflammation, hypertension, thrombosis, and possible involvement in COVID-19 pathogenesis. Resident macrophages, also called alveolar macrophages, are the first immune cells that might engage SARS-CoV-2 **(A)**. At the site of infection, migrating neutrophils and mast cells secrete CatG to the extracellular space and induce the expression of proinflammatory cytokines by macrophages. SARS-CoV-2 S protein binds to angiotensin-converting enzyme 2 (ACE2) on type II pneumocytes and myocardial cells **(B)**. Furin and transmembrane protease serine subtype 2 (TMPRSS2) prime the SARS-CoV-2 S protein for virus-cell fusion with the host cell membrane. Consumption of ACE2-expressing cells by SARS-CoV-2 infection causes an excessive activation of the renin angiotensin system (RAS). Angiotensinogen is converted by renin to angiotensin I (Ang I); Ang I is progressively digested to angiotensin II (Ang II) by the angiotensin-converting enzyme (ACE) and also by CatG. Ang II binds to the type I angiotensin receptor to induce vasoconstriction, hypertension, and inflammation. ACE2 digests Ang I to angiotensin 1-9 (Ang 1-9) and Ang II to angiotensin 1-7 (Ang 1-7), which binds to the Mas receptor and triggers vasodilation. CatG is able to degrade elastic fibers and the phospholipid transfer protein (PLTP) resulting in lung inflammation. Moreover, CatG can attract more macrophages and neutrophils at the site of inflammation, while lactoferrin (LF) increases the activity of CatG, which further activates platelets resulting in thrombosis and supports the development of atherosclerosis. Camostat and Nafamostat prevent trypsin-like serine protease TMPRSS2-mediated entrance of SARS-CoV-2 into the target cell. Whether boswellic acid, which inhibits CatG, interferes with the proteolytic activity of furin and TMPRSS2 needs to be clarified.

Phospholipid transfer protein (PLTP) regulates phospholipid transport in the blood circulation and is highly expressed within lung epithelial cells. Extracellular PLTP has been recognized to be low in BALF from patients suffering from COPD, due to the degradation of PLTP by CatG. Additionally, intranasal delivery of active CatG in mice induces inflammation by recruiting immune cells to the lung tissue and degradation of PLTP (Figure 2A); a blockade of PLTP destruction inhibits lung inflammation in COPD (Brehm et al., 2014). Moreover, hydrolysis of elastic fibers (elastin) by CatG and PR3 reduces the elasticity of the lung and represents a pathological feature of COPD as well as the development of emphysema (Gudmann et al., 2018).

The uncontrolled proteolytic activity of NE has been associated with numerous lung diseases (Polverino et al., 2017). In addition to the role of NE to clear phagocytosed bacteria and fungi in the pulmonary system, NE enhances the permeability of alveoli and capillaries as well as induces the formation of cytokines during ARDS. On the other hand, extensive proteolytic activity of NE in sepsis might lead to the destruction of glycocalyx (covers the pulmonary endothelium) (Suzuki et al., 2019). Additionally, NE levels can be used as a biomarker of bacterial infection in the progression of COPD (Thulborn et al., 2019). In cystic fibrosis patients, the numbers of neutrophils and levels of NE are significantly elevated in sputum (Sly et al., 2013). Moreover, NE and PR3, when secreted from activated neutrophils, can enter endothelial cells, and cleave NFκB to promote apoptosis (Preston et al., 2002). Increased catalytic activity of PR3 in COPD results in a high catalytic turnover of elastin in pulmonary parenchyma in relation to impaired lung elasticity and emphysema (Gudmann et al., 2018). Overall, the involvement of NSPs in chronic airway inflammation suggests that NSPs may be a potential drug target.

## General Aspects of Neutrophil Serine Proteases in Inflammation

NSPs regulate inflammatory processes via several means, including proteolytic truncation of chemokines, cytokines, and growth factors to modulate their activity, activation or shedding of cell-surface receptors at the sites of inflammation, and controlling pathways of apoptosis (Pham, 2008; Hahn et al., 2019). The recent research findings illustrate that CatG systematically degrades fibroblast growth factor-1 (FGF-1), IL-3, IL-6, IL-7, IL-15, IL-18, IL-31, and IL-33, stem cell factor (SCF), whereas NE and PR3 completely cleave cytokine ciliary neurotrophic factor (CNTF), connective tissue growth factor (CTGF), FGF-1, FGF-9, FGF-19, FMS-like tyrosine kinase 3 ligand (flt3L), granulocyte colony-stimulating factor (G-CSF), insulin-like growth factor-1 (IGF-1), IGF-2, IL-3, IL-7, IL-15, IL-16, IL-17A, IL-31, IL-33, neuregulin-1b, SCF, thymic stromal lymphopoietin (TSLP), but not IL-1-α, IL-5, IL-8, monocyte chemoattractant protein-1 (MCP-1), macrophage colony-stimulating factor (M-CSF), RANTES, TNF-α, which are resistant to proteolysis, proposing that NSPs selectively preserve a specific set of cytokines and chemokines necessary for recruitment of inflammatory cells to the sites of tissue damage or infection (Fu et al., 2017; Fu et al., 2020). Furthermore, PR3 can promote apoptosis in aging neutrophils via an activating cleavage of procaspase-3 in the cytosol of neutrophils. In contrast, PR3 proapoptotic activity can be blocked by SERPINB1 (Loison et al., 2014). NE, CatG, and PR3 can cleave and inactivate the C5a receptor which contributes to neutrophil dysfunction (van den Berg et al., 2014). Interestingly, NSP4 has been demonstrated to regulate storage of histamine and serotonin within secretory granules during early stages of mast cell development, which represents an essential component for mast cell-mediated vascular leakage and edema during allergic inflammation (AhYoung et al., 2020).

### Neutrophil Serine Proteases in Chronic Inflammation

NSPs are involved in various chronic inflammatory conditions. For instance, NE converts the IL-36 receptor antagonist to the mature active form, which in turn prevents IL-36-induced chemokine (IL-8 and CCL20) production by fibroblasts and keratinocytes, and thereby reduces psoriatic inflammatory cell infiltration (Macleod et al., 2016). IL-1β activation by NE within endothelial cells presumably promotes atherosclerosis (Alfaidi et al., 2015). Moreover, NE stimulates retinal vascular leakage during progression of diabetic retinopathy in a murine model most likely via the activation of myeloid differentiation primary response 88 (MyD88), NF-κB, and PAR2 as well as degradation of vascular endothelial cadherin (Liu et al., 2019). CatG has been found to be upregulated in colonic mucosa samples from patients with inflammatory bowel disease (IBD) proposing the involvement of CatG in IBD pathophysiology (Denadai-Souza et al., 2018). PR3, which is one of the major targets of autoantibodies in patients with granulomatosis with polyangiitis (GPA), binds to the plasma membrane of apoptotic cells, prevents the clearance of apoptotic cells by macrophages, interferes with common anti-inflammatory reprogramming of macrophages and initiates the synthesis of proinflammatory cytokines in macrophages, and blocks pDC-mediated induction of T regulatory cells. These conditions support systemic inflammation (Martin and Witko-Sarsat, 2017). NSPs are also reported to degrade NET proteins, which can be recognized by autoantibodies in patients with systemic lupus erythematosus and rheumatoid arthritis, and counteracting autoimmune reactions in relation to NET components (de Bont et al., 2020). Of note, NSPs are involved in the progression of diabetic cardiomyopathy, since genetic knockout of dipeptidyl peptidase I (DPPI, also known as cathepsin C), which is an essential protease for maturation of NSPs, prevents myocyte apoptosis and fibrosis development (Kolpakov et al., 2019).

### Interdependence of CatG and Lactoferrin Functions in Inflammation

LF is a ubiquitous, evolutionary ancient protein, playing a key role in the host’s defense by linking innate and adaptive immunity and maintaining homeostasis (Mayeur et al., 2016). Additionally, LF comprises the capability to reversibly bind two ferric (Fe^3+^) ions. LF is present at high concentrations in body excretions and on the mucous /membranes of eyes, the gastrointestinal duct, as well as the respiratory, and genital system. In these gates of infection, LF represents a very effective guardian, protecting the host against viral-, bacterial-, fungal-, and parasitic infections (Mayeur et al., 2016). Secondary granules of neutrophils embody another important reservoir of LF which can be released upon infection, locally or systemically, and act as alarmin (Kruzel et al., 2017). Degranulation of neutrophils at sites of infection and subsequent NET formation create a microenvironment promoting a fast and efficient killing and elimination of bacteria. In these processes one can envisage coordinated actions of the catalytic activity of enzymes from primary granules of neutrophils with LF. In fact, LF was found to augment at physiological concentrations enzymatic activity of CatG by lowering its specificity to the substrate and recovering CatG activity at a lower pH (Eipper et al., 2016). This phenomenon was validated by an additional study demonstrating an increase of protein turnover by CatG incubated with an excess of LF, and found that high serum levels of LF and CatG activity in osteomyelitis patients embody CatG polymorphism, characterized by the exchange of asparagine to serine at position 125 (N125S) (Thorpe et al., 2018). N125S polymorphism is associated with osteomyelitis and a lower survival rate following a cardiovascular or cerebrovascular episode (Herrmann et al., 2001; Perez-Is et al., 2019). Of interest, LF also possesses several enzymatic functions (Soboleva et al., 2019) and shares serine protease activity with CatG (Qiu et al., 1998). The protein was found to remove the IgA protease precursor from the outer bacterial membrane and degrade the serine protease autotransporter protein (Hap), preventing Hap-mediated adherence of bacteria to target cells. Suppression by serine protease inhibitors suggested that a fragment of the LF molecule in the N-lobe exhibits serine protease activity. In this way, two important invasive functions of bacteria can be eliminated. On the other hand, LF controls excessive antibacterial action of neutrophils by inhibition of NET formation (Okubo et al., 2016). This function of LF may be of particular importance in the process of CatG to activate endothelial cells by NETs (Folco et al., 2018). NETs accelerate clotting of re-calcified plasma and induce expression of VCAM-1 and ICAM-1. However, induction of adhesion molecules is inhibited by LF (Zimecki et al., 1999; Baveye et al., 2000). The activity of LF, released systematically upon infection, is evidently anti-inflammatory and, in theory, should counteract the proteolytic performance of CatG. Although both CatG and LF cause vasodilatation mediated by nitric oxide (Glusa and Adam, 2001; Garcia-Tejedor et al., 2017), in pathological conditions LF strongly inhibits nitric oxide production (Kruzel et al., 2002) leading to suppression of vasodilation. In general, CatG activates platelets, which can be further enhanced by the combined action of LF and CatG as was determined by an increase of the platelet activation marker CD62P (Eipper et al., 2016). Inversely, LF inhibits both the aggregation of platelets (Qian et al., 1995) and platelet production in mature megakaryocytes (Matsumura-Takeda et al., 2008). Thus, the consequence of LF on the formation of thrombus should be protective in venous thromboembolism, a fatal consequence in patients with severe COVID-19. The characteristics of heparin to modulate the activity of CatG are controversial and might depend on the heparin concentration (Ermolieff et al., 1994; Sissi et al., 2006; Fleddermann et al., 2012; Eipper et al., 2016). Additionally, the function of LF can be blocked by heparin, preventing the interaction of LF with the heparan sulfate proteoglycans, a molecule where SARS-CoV-2 is also able to attach to the host cell membrane (Hu et al., 2021). Considering interference of LF-dependent surveillance of CatG function with heparin, administered as an anti-coagulant, one may envisage application of other therapeutics (Bianconi et al., 2020). A therapeutic, oral application of recombinant human LF cannot be excluded since expression of antioxidant and anti-inflammatory genes are similarly upregulated by LF as upon intravenous treatment (Kruzel et al., 2021). In summary, proinflammatory functions of CatG in initiation of inflammation and infection (Brignone et al., 2001; Mezyk-Kopec et al., 2005) may be controlled by LF (Kruzel et al., 2017) with a particular reference to lung inflammation (Zimecki et al., 2021) induced by viruses or allergens.

### SARS-CoV-2, COVID-19, and Neutrophil Serine Proteases

Severe acute respiratory syndrome coronavirus 2 (SARS-CoV-2) is the causative agent responsible for the coronavirus disease 2019 (COVID-19) global outbreak. SARS-CoV-2 belongs to the genus *Betacoronavirus* in the *Coronaviridae* family (order *Nidovirales*), and is an enveloped positive-sense, single-stranded RNA virus. Epidemiological analysis of patients early in the epidemic indicates a zoonotic origin of SARS-CoV-2 (Gabutti et al., 2020; Zhu et al., 2020).

The SARS-CoV-2 S protein consists of the S1 subunit, which contains the N-terminal domain and a receptor-binding domain (RBD) with a receptor-binding motif (RBM), and the S2 subunit, covering the fusion peptide, heptad repeat 1, heptad repeat 2, a transmembrane domain, and a cytoplasm domain (Yuan et al., 2020). The S protein is the primary target for neutralizing antibodies to prevent viral infection, since SARS-CoV-2-RBD-specific IgG antibodies effectively block SARS-CoV-2-RBD binding to the host cell receptor human angiotensin-converting enzyme 2 (ACE2), a zinc metalloprotease that is also called peptidyl-dipeptidase (Turner et al., 2002; Chen et al., 2020).

The proteolytic cleavage of the S protein by mainly furin and the transmembrane protease serine subtype 2 (TMPRSS2) is a prerequisite for preparing the S protein to generate the fusion peptide. This is performed in a two-step sequential hydrolysis. First, the priming cleavage between the S1/S2 interface is performed by furin and is followed by an activating cleavage at the S2′ site by TMPRSS2 (Bestle et al., 2020). How is this achievable? After binding of the RBD within the S1 subunit to the cell surface ACE2, furin hydrolyzes the S1/S2 interface, containing the furin cleavage site (RRAR↓S), which might trigger a conformational change of the S2 subunit to predispose S2′ to be proteolytically digested by TMPRSS2 (Belouzard et al., 2009; Matsuyama et al., 2010; Bertram et al., 2013; Millet and Whittaker, 2014; Ou et al., 2016). After binding to ACE2, there are two possibilities regarding how the virus enters the host cell, namely directly or by using the receptor-mediated endocytosis pathway. The heptad repeats, within the S2’ subunit, insert into the cellular membrane and provoke membrane fusion to release the virus into the host cell cytoplasm. Alternatively, binding of the virus to ACE2 can lead to uptake of the virus into the host cell endosome, where the cysteine protease cathepsin L activates the S2 domain for delivery into the host cytoplasm (Simmons et al., 2013; Huang J. et al., 2020; Ou et al., 2020). S protein activation depends on both furin and TMPRSS2, which holds a promising target strategy for clinical management of COVID-19 (Hoffmann et al., 2020a; Bestle et al., 2020; Huang Y. et al., 2020; Ming and Qiang, 2020; Vankadari, 2020). Of note, 10% of the S protein (S1/S2 interface) of SARS-CoV-2 Wuhan is primed by furin, whereas more than 50% cleavage occurs for the Alpha variant, and over 75% for the Delta variant (Scudellari, 2021).

It is worth noting that two coagulation factors belonging to the serine protease class, namely factor Xa and thrombin, have been observed to activate the SARS-CoV-2 S protein for pseudoviral entry into Calu-3 and A549 cells, proposing that activation of coagulation cascade might exacerbate the infectious process (Kastenhuber et al., 2021).

In a recent study, the proteomic analysis of naso-oropharyngeal swap samples of SARS-CoV-2 patients showed neutrophil degranulation. Among others, elastase and CatG were significantly upregulated at the site of infection (Akgun et al., 2020). The imbalance between NE and anti-elastase activity of alpha-1 antitrypsin (A1AT) in the serum of patients admitted to intensive care (Zerimech et al., 2021) suggests a need to develop effective antiviral drugs to manage COVID-19 outcomes. NE and PR3 cleave the S protein of SARS-CoV-2 within the polybasic sequence of the proteolytically sensitive activation loop, suggesting a role in S protein priming by NSPs (Mustafa et al., 2021). An *in silico* analysis determined that the SARS-CoV-2 D614G substitution harbors a potential new NE cleavage site within the S protein (Bhattacharyya et al., 2021) and the SARS-CoV-2 Alpha variant harbors novel cleavage sites possibly between isoleucine 716 and asparagine 717 (716IN717) and between alanine 982 and arginine 983 (982AR983), and in the case of the SARS-CoV-2 Beta variant additional NE cleavages sites were proposed, increasing the propensity for NE to digest the S protein (Pokhrel et al., 2021).

### The Proteolytic Activity of Neutrophil Serine Proteases and the Cardiovascular System: A Possible Correlation to Severe Complications in COVID-19

COVID-19 patients with severe complications exhibit a significant decrease in lymphocyte counts, in contrast to higher neutrophil numbers, and the neutrophil-to-lymphocyte ratio or neutrophil-to-CD8^+^ T cell ratio are postulated to be predictive for the prognosis of severe cases of COVID-19 (Liu et al., 2020). Neutrophil blood counts, releasing mediators, and elevated NET generation of neutrophils in patients with COVID-19 might account for microvascular thrombosis in the lung, cytokine release, and respiratory failure (Zuo et al., 2020). Additionally, the excessive formation of NETs in COVID-19 patients leads to micro-vessel congestion and vascular damage (Leppkes et al., 2020) and an increased level of NE in the blood served as a predictor of disease severity in patients with SARS-CoV-2 infection (Gueant et al., 2021). Instead, NSPs are not proteolytically active in the formation of NETs and are released as inactive proteases (Kasperkiewicz et al., 2020). Thus, NET-derived NE might be impaired in the priming of the S protein of SARS-CoV-2 for host cell entrance.

Angiotensinogen is converted by renin to angiotensin I; angiotensin I is progressively digested to angiotensin II by the angiotensin-converting enzyme (ACE) but also by neutrophil-derived CatG and mast cell-derived chymase, as shown in Figure 2B. Angiotensin II binds to the type 1 angiotensin receptor to induce vasoconstriction and elevated blood pressure, kidney-mediated reabsorption of sodium and water, and enhance inflammation and fibrosis. The hydrolysis of angiotensin II (vasoconstrictor) to angiotensin 1–7 (vasodilator) by ACE2 is valuable in the renin angiotensin system (RAS) as angiotensin 1-7 physiologically stalls RAS (Kessler and Schunkert, 2020; South et al., 2020). Angiotensin 1-7 binds to the Mas receptor of diverse cell lineages of several tissues related to cardiovascular disease and lowers blood pressure by vasodilation, causes kidneys to excrete sodium and water, and attenuates inflammation. The balance between both pathways controls the outcome to a stimulus (South et al., 2020). Binding of SARS-CoV-2 to ACE2 subsequently downregulates cell surface expression of ACE2, resulting in angiotensin II accumulation, local RAS activation, and organ injury (Vaduganathan et al., 2020). It has been suggested that patients with severe COVID-19 have a history of comorbidities, including cardiovascular diseases, chronic kidney disease, and type 2 diabetes mellitus (Zhou F. et al., 2020; Pitocco et al., 2020).

Excess of the catalytic activity of CatG is one of the bases of cardiovascular and concomitant inflammatory comorbidities. Inhibition of CatG activity is a plausible strategy for a possible treatment (Korkmaz et al., 2010; Ortega-Gomez et al., 2016). So far, four polymorphisms in human CatG are described, three of which have no effect on promotor- or transcription activity, and the fourth, present in the coding region (N125S), is linked to an accumulation of plasma fibrinogen in patients which might be due by the point that CatG ensures the release of fibrinogen (Rabhi-Sabile et al., 1996; Herrmann et al., 2001). Fibrinogen contributes to atherosclerosis, a coronary event, and strokes (Fuster et al., 1990; Lee et al., 1993) indicating a lower survival rate after a cardiovascular or cerebrovascular episode in these patients (Herrmann et al., 2001). It can be speculated that N125S polymorphism might change the proteolytic activity or the substrate specificity of CatG, thereby increasing the activation of platelets. However, there is no difference in CatG N125S polymorphism regarding infections associated with sepsis (Sipahi et al., 2006). Whether the observation of the outcome of severity in COVID-19 might be related to CatG or polymorphisms of CatG is not known.

### Bradykinin Storm

In the respiratory system, migrating neutrophils and mast cells secrete, among others, CatG, which shows the ability to provoke the expression of proinflammatory cytokines in macrophages (Moriuchi et al., 2000; Maryanoff et al., 2010). The so-called cytokine storm, mediated by alveolar macrophages and induced by SARS-CoV-2 infection, might be the major reason for a severe outcome of COVID-19 (Tay et al., 2020). Notwithstanding, recent findings have challenged the cytokine storm hypothesis (or rather extended) and suggest that the pathophysiology of COVID-19 is related to bradykinin storm and elevated hyaluronic acid levels that cause hydrogel formation in the lungs and thereby interfere with the normal gas exchange process (Garvin et al., 2020). Bradykinin (RPPGFSPFR 1-9), produced from kininogen by kallikrein, activates bradykinin receptor B2 to promote vasodilation, hypotension, and vascular permeability. Additionally, angiotensin 1-9 is able to augment this signaling pathway. Bradykinin (RPPGFSPF 1-8), which is liberated from bradykinin by carboxypeptidase N, acts on bradykinin receptor B1 to induce a pain response, vascular leakage, and neutrophil recruitment to sites of inflammation (Garvin et al., 2020). The recruitment and activation of neutrophils initiate the secretion of CatG, inducing endothelium-dependent vascular relaxation (Glusa and Adam, 2001). Modulation of the bradykinin signaling pathway is possible by CatG as well as kallikrein, with both being able to directly activate the bradykinin receptor B2 (Hecquet et al., 2000). More importantly, CatG (also chymase) has been shown to cleave bradykinin at the carboxy terminal end between 8FR9 generating bradykinin RPPGFSPF 1-8. Interestingly, CatG does not digest between 5FS6, based on the fact that CatG does not hydrolyze the peptide bond when proline is at P3 position (Reilly et al., 1985). This supports the notion that CatG might also be involved in the bradykinin storm.

## Protease Inhibitors for Possible Clinical Management of Patients

After the release of NSPs by activated neutrophils into the inflamed tissue, these proteases have to be tightly regulated to avoid serious complications. The presence of endogenous serine protease inhibitors in the extracellular space regulates the catalytic activity of NSPs. As an example, the α1-proteinase inhibitor interferes with NE, PR3, NSP4, and CatG activity, α1-anti-chymotrypsin inhibits CatG, and secretory leukocyte protease inhibitor impairs CatG activity (Korkmaz et al., 2010). Under physiological conditions the regulation of proteases is not always likely. The imbalance between the activity of NSPs and their endogenous inhibitors can cause chronic inflammatory disorders (Korkmaz et al., 2020). Therefore, inhibition of serine proteases is a promising therapeutic strategy to interfere with the development of serious diseases (Kosikowska and Lesner, 2013; Dey et al., 2018) and will be discussed for NSPs in the following sections.

### Inhibition of the Proteolytic Activity of CatG

**FIGURE 3:**
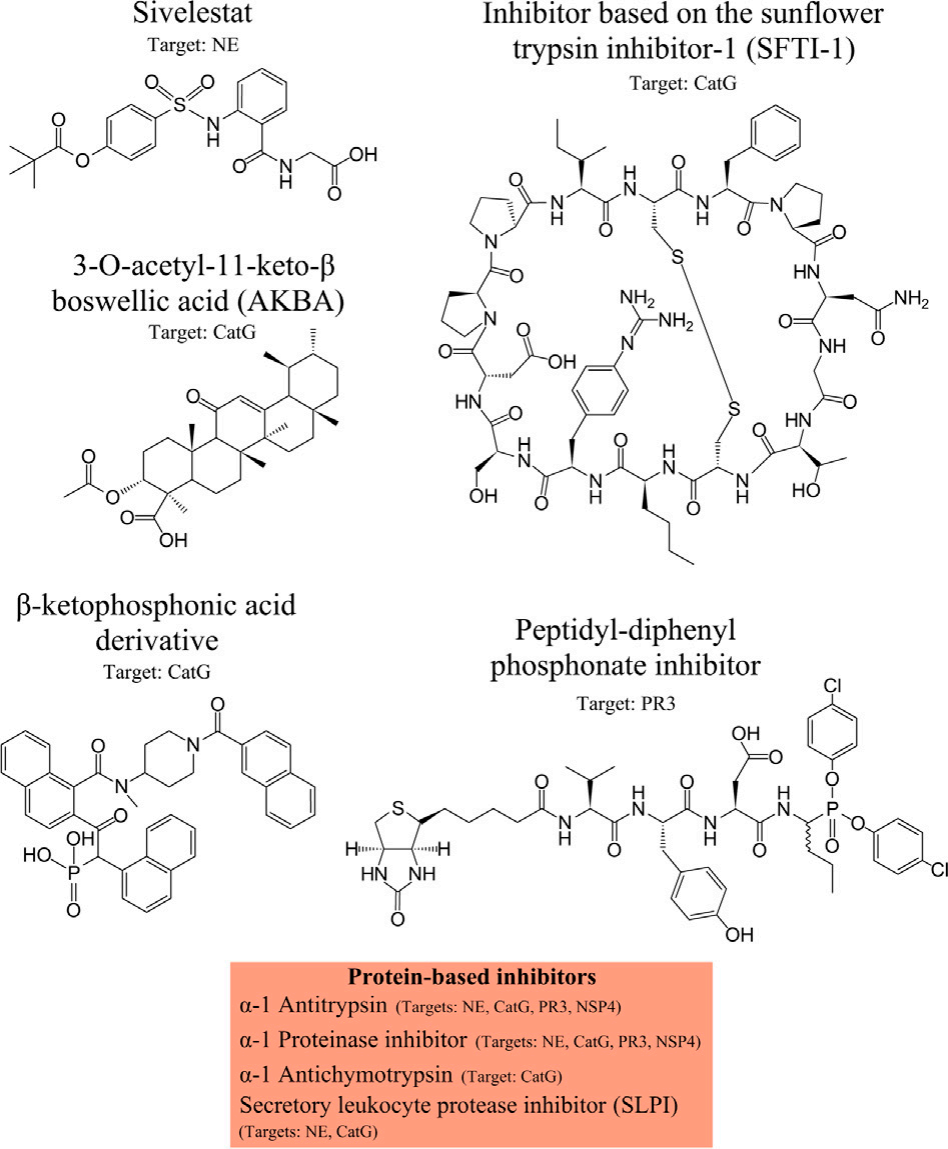
NSPs inhibitors. Neutrophil serine protease (NSP) inhibitors with their specific targets are shown from left to the right: Sivelestat, inhibitor based on the sunflower trypsin inhibitor-1 (SFTI-1) scaffold, 3-O-acetyl-11-keto-β boswellic acid (AKBA), β-ketophosphonic acid derivative, peptidyl-diphenyl phosphonate inhibitor, and protein-based inhibitors, including α-1 antitrypsin, α-1 proteinase inhibitor, α-1 antichymotrypsin, secretory leukocyte protease inhibitor (SLPI). The main protease targets are indicated.

Therapeutic inhibitors of CatG are available and are sub-divided into two major groups: Peptide- or protein-based inhibitors and non-peptide inhibitors. The first group of CatG inhibitors consists of bovine pancreatic trypsin inhibitors, alpha-1 anti-chymotrypsin, alpha-1 antitrypsin, template based β-hairpin peptidomimetics, chymostatin, and alpha-aminoboronic acid peptides. The second group of CatG inhibitors includes organophosphorus inhibitors such as diphenyl esters of (alpha-aminoalkyl) phosphonates and β-keto-phosphonic acid derivatives, polysaccharides such as 2-*O*-desulphated heparin and synthetic dextran derivatives, heterocyclic compounds (thiazolidines), aptamers, and boswellic acid derivatives (extensively reviewed in (Kosikowska and Lesner, 2013)) (Figure 3).

CatG activity can be inhibited by boswellic acids (BAs) (constitutes of frankincense, IC_50_ of 0.6 μM) in a competitive and reversible manner. In a functional assay, BAs perturbs chemoinvasion of neutrophils and oral administration of BA significantly attenuates CatG activity in human blood. Of the four BA variants, 3-O-acetyl-11-keto-β boswellic acid (AKBA), α- and β-boswellic acids, as well as 11-keto-β-boswellic acid, AKBA is the most potent BA derivative in suppressing inflammation (Tausch et al., 2009; Abdel-Tawab et al., 2011). BA displays anti-inflammatory, cardio-, neuro-, and hepatoprotective, analgesic as well as anti-cancer, -microbial, -diabetic, and -thrombotic capacity. Moreover, BA decreases invasion of neutrophils, reduces differentiation of T effector cells as well as enhances differentiation of T regulatory cells, and diminishes infiltration of immune cells in inflamed tissues (Efferth and Oesch, 2020). These observations fit the findings that CatG is upregulated in inflammation and chronic inflammatory pain (Liu et al., 2015). Additionally, as we demonstrated in immune cells of type 1 diabetes (T1D) patients, high levels of CatG were found and were responsible for insulin antigen processing as well as antigen presentation to activate autoreactive T cells, which can be prevented by the CatG inhibitor (Zou et al., 2011). Whether BA also disturbs the catalytic activity of TMPRSS2 or blocks the migration of activated neutrophils to the lung tissue in severe cases of COVID-19 would be interesting to investigate.

Sunflower-derived serine protease inhibitors are potent and selective CatG inhibitors. These are engineered components based on a 14 amino acid cyclic peptide with a head-to-tail cyclization, bisected by a disulfide bond, which are stable from hydrolysis and are referred to as sunflower trypsin inhibitor-1 (SFTI-1, GRCTKSIPPICFPD, K represents P1 position for CatG cleavage) (Legowska et al., 2009; Lesner et al., 2011). Substitution of lysine at position 5 to phenylalanine within the SFTI-1 sequence improved the inhibitory capacity towards CatG. An additional replacement of an amino acid at this position through the incorporation of 4-guanidyl-L-phenylalanine further increased the selectivity of the compound (Legowska et al., 2009). Meanwhile exchanging amino acids at preferred CatG cleavage sites, as well as integrating norleucine, and 4-guanidyl-L-phenylalanine, resulted in an extremely potent CatG inhibitor (component 22, GTCnXSDPPICFPN, n = norleucine, X = 4-guanidyl-L-phenylalanine, in bold replaced amino acids) with constant subnanomolar inhibition (*K*i = 1.6 nM±0.2 nM) and a very high selectivity over other serine proteases which have tryptic and chymotryptic specificity (Swedberg et al., 2017). This potent and specific CatG inhibitor might also be promising for inhibition of CatG in pathophysiological conditions, such as autoimmunity, chronic inflammatory disorders, cardiovascular diseases, and thrombosis.

### Neutrophil Elastase Inhibitors

Taking into consideration that the level of natural NE inhibitors remains diminished and NE activity is elevated in cardiovascular and pulmonary diseases, such as ARDS, COPD, and acute lung injuries, engineered NE inhibitors are tested for potential treatment options (von Nussbaum et al., 2015; Polverino et al., 2017). For instance, Emodin (a NE inhibitor) suppresses pulmonary fibrosis (Zhou L. et al., 2020). Sivelestat, which is another NE inhibitor, reduces lung injury and the survival rate, decreases the level of IL-8 (neutrophilic chemoattractant), and significantly decreases the number of neutrophils in BALF samples in comparison to control mice (Mikumo et al., 2017). An additional study has reported that Sivelestat reduces the content of pleural effusion as well as levels of albumin (a marker of lung permeability), decreases myeloperoxidase activity (indicating neutrophil infiltration), amplifies oxygenation, and improves the survival rate. Pre-treatment with Sivelestat perturbs the development of lung edema and prevents neutrophil accumulation in mice with ventilator-induced lung injury (VILI) that is characterized by a neutrophil-predominant inflammatory response, vascular leak, and alveolar damage (Kim et al., 2014). In mice treated with LPS to cause an endotoxemia and subsequent myocardial injury, administration of Sivelestat prompted a higher survival rate by reduction of pro-inflammatory IL-6 and decreased neutrophil infiltration (Fukuta et al., 2020). Sivelestat was identified to be the most beneficial for patients with mild ARDS (Tagami et al., 2014; Maki et al., 2020). Additionally, application of Sivelestat is valuable for patients who underwent a cardio-pulmonary bypass that induces post-pump syndrome. The post-pump syndrome describes a systemic inflammation due to accumulation of neutrophils and release of NE among other mediators. Treatment of patients positioning for minimal invasive cardiac surgery with Sivelestat prevents the development of post-operative lung injury and contributes to the reduction of ventilation time and hospital stay (Yamashiro et al., 2018). Remarkably, inhaled Sivelestat is predominantly recommended since Sivelestat can directly reach the lung tissue (Tao et al., 2012). The combination of non-invasive ventilation and a NE inhibitor was documented to improve the survival ratio for mild and moderate ARDS patients, demonstrating the advantage of combinatorial therapy to avoid endotracheal intubation and subsequent mechanical ventilation (Tsushima et al., 2014). Notably, NE in patients with cystic fibrosis can destroy the lung architecture by degradation of mucin which in turn ensures lung dysfunction. As a rational result, the application of KRP-109, a selective NE inhibitor, is recommended for a potential procedure to treat cystic fibrosis, because KRP-109 significantly delayed mucin degradation in the sputum of cystic fibrosis patients (Chillappagari et al., 2016). POL6014, another selective NE inhibitor administrated by inhalation, effectively inhibited NE in BALF and sputum samples of patients with cystic fibrosis *ex vivo* (Barth et al., 2020). Moreover, a highly selective and competitive inhibitor of NE, BAY85-8501 (von Nussbaum et al., 2015), implies the management of patients with non-cystic fibrosis bronchiectasis with BAY85-8501 (Watz et al., 2019). Taken together, NE inhibitors present a potent means to treat patients with cardiovascular and pulmonary diseases in cases where NE activity is not properly regulated.

### PR3 Inhibitors

PR3 digests elastin in lungs and extracellular matrix components, such as fibronectin, laminin, vitronectin, and collagen type IV (Rao et al., 1991). On the contrary, uncontrolled PR3 can lead to considerable tissue damage and emphysema (Rao et al., 1991), which may further turn into chronic obstructive pulmonary disease (Gudmann et al., 2018). In addition, PR3 activates PAR2, which can result in inflammation and triggering the onset of arthritis, colitis, and airway diseases (Novick et al., 2006).

In the case of bound PR3 to the cell surface, PR3 can be recognized by anti-neutrophil cytoplasmic antibodies (ANCA) (Maximova et al., 2019). This binding results in neutrophil activation leading to inflammation and different pathological conditions such as ANCA-associated vasculitis, including granulomatosis with polyangiitis (Wegener's granulomatosis), (Matsumoto et al., 2008). Similar to other neutrophil serine proteases, PR3 is regulated by natural serine inhibitors, such as alpha2-macroglobulin, the alpha-1 protease inhibitor, monocyte neutrophil elastase inhibitors, elafin, and the secretory leukocyte protease inhibitor (Grzywa et al., 2019). However, PR3 was found to be comparatively resistant to the alpha-1 protease inhibitor, secretory leukocyte protease inhibitor (Grzywa et al., 2019) as well as to alpha 2-macroglobulin (N'Guessan et al., 2020). The alpha-1 protease inhibitor is not able to interfere with the binding of ANCA to membrane-bound PR3 (Maximova et al., 2019). Moreover, alpha1-antitrypsin deficiency (AATD) can lead to the development of COPD (Dunlea et al., 2018). AATD patients can be treated with AAT augmentation therapy which results in decreased levels of AαVal541, a peptide marker generated by the cleavage of fibrinogen by PR3 (Schouten et al., 2021). Infusion of AAT negates the activity of PR3 and prevents the development of lung emphysema in AATD. In another cohort study, patients with type 2 diabetes and non-alcoholic fatty liver disease had elevated levels of PR3 (Mirea et al., 2019). Therefore, administration of AAT egresses the management of AATD. Furthermore, the expression of membrane-bound PR3 is higher in patients with rheumatoid arthritis, whereas the cell surface expression of PR3 is further upregulated in the presence of pro-inflammatory cytokines, since PR3 proteolytically cleaves TNF-α to the active TNF-α form (Matsumoto et al., 2008). These findings indicate that PR3 plays a specific role in maintaining the inflammatory environment in rheumatoid arthritis. Patients who received infliximab (monoclonal antibody binding to TNF-α) exhibited lower membrane-bound PR3 and their overall condition was improved (Matsumoto et al., 2008). Similarly, rituximab (binding to CD20 of B cells) has the potential to be used for treatment of PR3-ANCA associated diseases (Yoshida et al., 2021) and PR3-ANCA-associated with granulomatosis with polyangiitis, rituximab was observed to maintain the remission with non-adverse effects (Eriksson, 2005). Contrastingly, in another study, rituximab resulted in remission of PR3-ANCA-associated vasculitis, where several patients established complications, such as infection and hepatitis B reactivation (Wendt et al., 2012). An *in vitro* study suggested the use of INS1007, a reversible dipeptidyl peptidase-1 inhibitor, to reduce cell surface levels of PR3 on neutrophils, since dipeptidyl peptidase 1 is essential to convert pro-NSPs to their proteolytically active form (Zhang et al., 2019). More investigations are needed to determine whether INS1007 can be implemented for treatment of PR3-mediated diseases.

### NSP4 Inhibitors

The catalytic activity of NSP4 is not impaired by most of the natural serpins. Excess of alpha-1 protease inhibitor, antithrombin with heparin, and C1 inhibitor hinder the activity of NSP4. Among chemical inhibitors, NSP4 is strongly inhibited by H-D-Phe-Pro-Arg-chloromethylketone and by the non-selective serine protease inhibitor phenylmethylsulfonyl fluoride (Perera et al., 2012).

### Serine Protease Inhibitors and Interference With SARS-CoV-2 Infection

**FIGURE 4:**
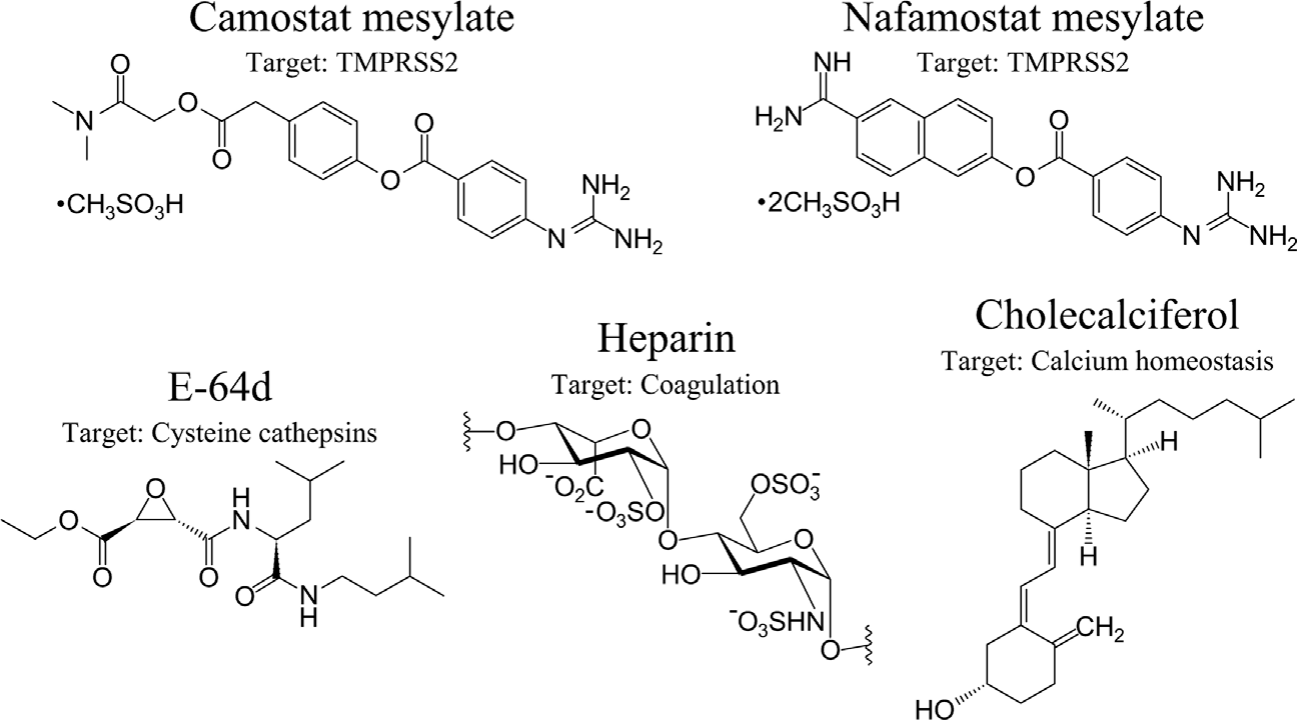
Therapeutic option in the context of SARS-CoV-2 and COVID-19. Anti-SARS-CoV-2 therapeutic options with specific targets are presented from left to the right: Camostat, Nafamostat, aloxistatin/E-64d, heparin, and cholecalciferol (vitamin D_3_). Their major protease targets are indicated. Protein-based inhibitors, including α-1 antitrypsin and aprotinin/bovine pancreatic trypsin inhibitor, are nlot shown.

Hydrolysis of the SARS-CoV-2 S protein (S2’) by TMPRSS2 can be decisively blocked by Camostat mesylate (Camostat), which is a clinically proven trypsin-like serine protease inhibitor and previously used for the treatment of chronic pancreatitis (Ohshio et al., 1989; McKee et al., 2020) (Figure 4). Furthermore, Camostat and E-64d, a cell permeable inhibitor for cysteine proteases, referred to as cathepsin L, decline SARS-CoV-2 S protein-driven pseudotyped SARS-CoV-2 infection of the host cell and incubation of lung cells by Camostat is suggested to inhibit the entry of SARS-CoV-2 to the target cell (Hoffmann et al., 2020b). Both trypsin-like serine protease inhibitors Camostat and Nafamostat inhibit the proteolytic activity of TMPRSS2, while Nafamostat is 15 times more effective at interfering with host entry of SARS-CoV-2 compared to Camostat (Hoffmann et al., 2020b; Hoffmann et al., 2020c). The rationale is that Camostat together with its structural analogue Nafamostat, and other serine protease inhibitors, specifically aerosolized aprotinin, could prevent serine protease-mediated priming of the SARS-CoV-2 S protein (Hoffmann et al., 2020b; Stopsack et al., 2020). Therapeutically achievable concentrations were reached with aprotinin to interfere with SARS-CoV-2 replication in the target cell (Bojkova et al., 2020). Naturally occurring A1AT decreases acute lung injury by inhibiting inflammation, coagulation, and the catalytic activity of NSPs and TMPRSS2. A1AT is an attractive component for COVID-19 care (de Loyola et al., 2020; Yang et al., 2020) but aprotinin might be more effective (Bojkova et al., 2020). Contrastingly, aprotinin has antifibrinolytic features, causing serious adverse cardiovascular, renal, and cerebrovascular results (Lien and Milbrandt, 2006; Mangano et al., 2007), which might impair the benefits of aprotinin. Although Camostat is a promising drug and is well tolerated in COVID-19 patients (Gunst et al., 2021), in a randomized, double-blind, placebo-controlled clinical trial, Camostat did not elicit any benefits to manage COVID-19 patients. In reverse, it cannot be excluded that COVID-19 patients might benefit when Camostat is administered at the beginning of an infection (Gunst et al., 2021) or possibly with a combination of different inhibitors targeting the catalytic activity of proteases involved in comorbidities, including COVID-19. In this regard, it would be beneficial if Nafamostat also impairs the proteolytic activity of CatG (secreted or bound on the cell surface of neutrophils), thereby interfering with severe complications of COVID-19, but this hypothesis needs to be elucidated.

Moreover, NE could be a promising target for COVID-19. A1AT is a glycoprotein that originates in the liver and circulates in the blood. One of the principal functions of A1AT is to protect lung connective tissue by inhibiting NE in the lower respiratory tract (Topic et al., 2018) and can be implemented as a treatment of COVID-19. Besides the protease inhibitory ability, A1AT comprises also an anti-inflammatory function, reducing the cytokine storm by regulating IL-1β, IL-6, IL-8 production (McElvaney et al., 2021) and decreasing cell death as well as the formation of NETs (Yang et al., 2020). A1AT directly binds to NE and forms an A1AT-NE complex, inhibiting NE activity (McElvaney et al., 2021). A recent study has revealed that patients infected with SARS-CoV-2 showed low levels of functional A1AT and elevated levels of truncated A1AT, which contributes to the development of ARDS; a complication of COVID-19 (Yang et al., 2020). Furthermore, oxidation of Met358 and Met351, located in the active site of A1AT, reacts to methionine sulfoxide that reduces A1AT’s inhibitory activity, decreasing the second order association rate constant of NE (Topic et al., 2018). As a result, low level A1AT causes an imbalance between NE and A1AT, which probably leads to the development of COPD (Topic et al., 2018). Moreover, A1AT is a potential treatment option for patients with cystic fibrosis that are infected with SARS-CoV-2 (McElvaney et al., 2021). Some of these patients suffer from chronic infection by *Pseudomonas aeruginosa* and *Stenotrophomonas maltophila*; *Stenotrophomonas maltophila* might contribute to the entry of SARS-CoV-2 by degrading cell tight junction proteins ZO-1 and occludin. Furthermore, cystic fibrosis patients have increased NE activity with elevated levels of pro-inflammatory cytokines IL-1β, IL-6, IL-8 (McElvaney et al., 2021). In addition, Nasoil, a drug that consists of *Asclepias currasavica* extracts, modifies the elastic proteins of the respiratory system, improving the lung function, and controlling COVID-19 symptoms (Cuevas-Barragan et al., 2020). The severity of SARS-CoV-2 is also assumed to be associated with a “proteolytic storm” induced by neutrophils and NETosis (Thiam et al., 2020). Administration of Sivelestat is promising to interfere with ARDS progression (Tagami et al., 2014). Sivelestat improved the disseminated intravascular coagulation score and survival rate of patients (Hayakawa et al., 2010), and considerably downregulated the NE-mediated chemotaxis and inflammatory modulators, namely TNF-α, IL-6, and high mobility group box 1 (Hagiwara et al., 2009). Thus, selective inhibition of NE could substantially improve COVID-19 prognosis (Sahebnasagh et al., 2020).

### Heparin

In general, heparin is clinically administered as an anti-coagulant and particularly the low-molecular-weight form shows an anti-thrombotic feature. The highly sulfated glycosaminoglycan heparin encompasses negatively charged sulfate groups along with the polymer and can bind positively charged molecules (Kjellen and Lindahl, 1991), which should be kept in mind regarding the off-target effects of heparin. The mechanism of anti-coagulants is achieved by the heparin-mediated allosteric induced conformation change of antithrombin III for activation. In turn, antithrombin III inhibits thrombin, and, as a result of this inhibition, interference with the coagulation cascade and activation of platelets occurs (Evangelista et al., 1992; Walker and Royston, 2002). Indeed, thromboembolism was determined in systematic post-mortem examinations of COVID-19 patients (Wichmann et al., 2020); therefore, treatment of the pathogenesis of COVID-19 with low-molecular-weight heparin is recommended, which also reduces IL-6 (Perna et al., 2020; Thachil, 2020). Remarkably, in a U.S. cohort study, no mortality benefits between administration of therapeutic and prophylaxis doses of heparin were determined (Lynn et al., 2021) and the probability of venous thromboembolism remains high despite the thromboprophylaxis (Pizzolo et al., 2020). Besides heparin as a blood thinner to promote anticoagulation via binding to anti-thrombin III, heparin is also beneficial in COVID-19 by preventing endothelial leakage (inhibition of heparanase) and blocking leukocyte infiltration (Buijsers et al., 2020). Strikingly, a retrospective study of COVID-19 patients in Italy outlined that intermediate administration of low molecular weight heparin for 7 days is associated with a lower mortality rate (Paolisso et al., 2020). In an observational study of COVID-19 patients, low molecular weight heparin was associated as a protective factor since there was a reduced risk of the progression of ARDS (Falcone et al., 2020). Notwithstanding the promising available data, the number of COVID-19 patients that did not benefit from heparin remains high and the risk of bleeding events is significant (Daughety et al., 2020; Thomas et al., 2020).

The ability of heparin to inhibit or activate CatG is the subject of controversy. According to an early report by Ermolieff et al., the binding of heparin fragments to CatG causes steric hindrance to the access of α1-antichymotrypsin, α1-proteinase inhibitor, and eglin c and mitigates CatG activity (Ermolieff et al., 1994). On the molecular level, heparin engages with CatG by non-competitive inhibition (Sissi et al., 2006). In contrast to another study, CatG activity was significantly inhibited only in a low concentration of heparin (Fleddermann et al., 2012). Similarly, we found that an excess of heparin enhances CatG activity (Eipper et al., 2016). The concentration of heparin influences the proteolytic activity of CatG. Heparin competitively inhibits NE by binding tightly to the active site (Spencer et al., 2006). Additionally, heparin exhibits a strong affinity to NSP4 and the activity of NSP4 was significantly inhibited by antithrombin with heparin (Perera et al., 2012; Lin et al., 2014). These studies suggest that administration of heparin in a concentration dependent manner inhibits the activity of CatG, NE, and NSP4.

### Vitamin D

It is well known that 1,25 (OH)_2_D3 (vitamin D) can modulate immune cells, for instance, we found that vitamin D as well as a CatG inhibitor impair the activation of proinsulin-reactive T cells (auto-reactive T cells) from T1D patients, because CatG is elevated in peripheral blood mononuclear cells from T1D patients and enhances insulin antigen processing (Zou et al., 2011). Another study pointed out that inhibition or downregulation of CatG in non-obese diabetic mice reduced the activation of CD4^+^ T cells, improved the function of insulin-secreting β cells in the pancreas, and slowed down the progress of diabetes (Zou et al., 2017). Furthermore, human conventional DCs treated with vitamin D downregulate CatG activity (Zou et al., 2011), which might be due to the vitamin D-mediated upregulation of serine protease inhibitors (Burster et al., 2020) as have been observed for CD11c^+^ bone marrow-derived mouse DCs just recently (Saul et al., 2019). Interestingly, T1D-derived cDCs and their endocytic resident CatG resist inhibition by vitamin D (Zou et al., 2011). Importantly, vitamin D deficiencies are associated with more severe cases of COVID-19, since vitamin D inhibits the production of the aspartic protease REN, also known as angiotensinogenase, which converts angiotensinogen to angiotensin I; therefore, supplementation of vitamin D could be beneficial for COVID-19 patients (Garvin et al., 2020). It is worth speculating that an autoimmune pathophysiological response and defects in vitamin D-mediated upregulation of serine protease inhibitors could also lead to a severe case of COVID-19.

### Perspectives

Priming of the SARS-2 S protein is an essential step in the process of viral entry, which can be efficiently blocked by serine protease inhibitors. Venous thromboembolism might be the main cause of death in patients with severe COVID-19, indicating that the treatment of severe cases with low-molecular-weight heparin, even though it should be used with caution (rare cases of heparin-induced thrombocytopenia), is a logical course of action. Tissue damage is mediated by an excess of proteolytic activity of different proteases of neutrophils and timing is crucial for the effectiveness of treatment. While Sivelestat might interfere with the impact of SARS-CoV-2 infection of host cells, blood thinners and anti-inflammatory drugs are beneficial to interfere with a severe outcome of COVID-19 (a summary is illustrated in Figure 2). Additionally, various serine protease inhibitors pose a promising therapeutic approach for different cardiovascular and inflammatory diseases.

## Conclusion

The proteolytic activity of NSPs mediates numerous processes in physiological and pathophysiological conditions. NSPs, secreted from activated neutrophils as part of natural immunity, regulate various conditions related to cardiovascular, pulmonary systems as well as inflammation. However, uncontrolled proteolytic activity of NSPs can trigger conditions that are characterized by increased hydrolysis of targets. In such cases, the application of protease inhibitors might substantially improve the clinical outcomes. Therefore, the administration of inhibitors for clinical management of certain diseases is indicated.

## Glossary

A1AT: alpha-1 antitrypsin
AATD: alpha1-antitrypsin deficiency
ACE2: angiotensin-converting enzyme 2
AKBA: 3-O-acetyl-11-keto-β boswellic acid
ANCA: anti-neutrophil cytoplasmic antibodies
APCs: antigen-presenting cells
ARDS: acute respiratory distress syndrome
BA: boswellic acid
BALF: bronchoalveolar lavage fluid
BAX: Bcl-2 associated X
CAD: coronary artery disease
Cat: cathepsin
CatG: cathepsin G
CNTF: cytokine ciliary neurotrophic factor
COPD: chronic obstructive pulmonary disease
COVID-19: coronavirus disease 2019
CTGF: connective tissue growth factor
DPPI: dipeptidyl peptidase I
EGF: epidermal growth factor
FGF: fibroblast growth factor
flt3L: FMS-like tyrosine kinase 3 ligand
G-CSF: granulocyte colony-stimulating factor
GPA: granulomatosis with polyangiitis
IBD: inflammatory bowel disease
ICAM-1: intercellular adhesion molecule 1
IGF-1: insulin-like growth factor
IgG: immunoglobulin G
IL: interleukin
LF: lactoferrin
LPS: lipopolysaccharides
MCP-1: monocyte chemoattractant protein-1
M-CSF: macrophage colony-stimulating factor
MMPs: matrix metalloproteinases
MyD88: myeloid differentiation primary response 88
NE: neutrophil elastase
NETs: neutrophil extracellular traps
NF-kB: nuclear factor kappa-light-chain-enhancer of activated B cells
NK cells: natural killer cells
NSP4: neutrophil serine protease 4
PAH: pulmonary arterial hypertension
PAR: protease-activated receptor
pDC: plasmacytoid dendritic cells
PLTP: phospholipid transfer protein
PR3: proteinase 3
RAS: renin angiotensin system
RBD: receptor-binding domain
RBM: receptor-binding motif
SARS-CoV-2 S protein: severe acute respiratory syndrome coronavirus 2 spike protein
SCF: stem cell factor
SFTI-1: sunflower trypsin inhibitor-1
T1D: type 1 diabetes mellitus
TGF-β: transforming growth factor beta
Th: T helper cells
TMPRSS2: transmembrane protease serine subtype 2
TNF-α: tumor necrosis factor α
TSLP: thymic stromal lymphopoietin
VCAM-1: vascular cell adhesion protein 1
